# Current status of vision and refractive errors among children and adolescents in northeast Sichuan, China: a school-based cross-sectional study

**DOI:** 10.3389/fmed.2026.1798513

**Published:** 2026-05-13

**Authors:** Zhe Li, Ximin Yin, Xinyi He, Wenchuan Liao, Yunchun Zou

**Affiliations:** 1 Department of Ophthalmology, the Second Clinical College of North Sichuan Medical College (Nanchong Central Hospital), Nanchong, Sichuan, China; 2 Department of Optometry & Ophthalmology, North Sichuan Medical College, Nanchong, Sichuan, China; 3 Department of Ophthalmology, Affiliated Hospital of North Sichuan Medical College, Nanchong, Sichuan, China

**Keywords:** children and adolescents, cross-sectional study, northeastern Sichuan, refractive errors, visual acuity

## Abstract

**Purpose:**

To investigate the status of visual acuity and refractive errors among children and adolescents in northeastern Sichuan, providing a basis for targeted myopia prevention and control.

**Methods:**

In 2024, a school-based vision screening program using a stratified cluster sampling framework was conducted among students from participating schools in 3 districts and 6 counties of Nanchong City. Visual acuity screening and non-cycloplegic autorefraction were performed using an electronic intelligent screening system with real-time data collection and strict quality control. Of the 61,012 students initially screened, 59,319 students with complete and analyzable data were included in the final analysis, corresponding to an effective screening rate of 97.23%. Detection rates and distribution characteristics of poor visual acuity and suspected myopia were analyzed, along with associated risk factors.

**Results:**

The prevalence of poor visual acuity and suspected myopia increased markedly with educational stage, reaching 90.81% and nearly 90%, respectively, in general senior high school students, accompanied by a parallel increase in myopia severity and high myopia proportion. Suspected myopia was more common among female students. Substantial regional disparities were observed, with the highest prevalence in Nanbu County (80.49%) and the lowest in Langzhong City (46.41%). Urban students had significantly higher myopia prevalence than township students. In addition, during the general senior high stage, students in ordinary schools showed significantly higher myopia prevalence than those in key schools. Regarding vision correction, eyeglasses were the predominant method. The rate of spectacle use increased with education level, though proper correction remained suboptimal. Additionally, astigmatism and anisometropia were prevalent among myopic students and increased with school grade. Hyperopia reserve declined progressively in lower-grade students. Multivariable logistic regression analysis showed that school grade, gender, regional type, and school level were independently associated with myopia.

**Conclusions:**

This study reveals the epidemiological characteristics and influencing factors of visual acuity and refractive errors among children and adolescents in northeastern Sichuan, providing essential data and scientific evidence for the development of targeted myopia prevention strategies in the region.

## 1 Introduction

Myopia has emerged as a major global public health challenge threatening the visual health of adolescents. Its widespread prevalence and profound impact have drawn increasing international attention (1, 2). In China, the situation is particularly severe, with persistently high myopia rates among adolescents and an alarming trend toward younger onset (3, 4). According to national statistics, the overall myopia prevalence among Chinese children and adolescents reached 53.6% in 2018 (5). Myopia can directly impair learning efficiency, cause visual blurriness and reading difficulties, restrict participation in outdoor activities and physical exercise, and even lead to serious ocular complications such as retinal detachment and macular degeneration in high myopia, posing a lifelong threat to visual health (6–8).

China has elevated adolescent myopia prevention to a national strategic priority, releasing a series of policy documents and guidelines to coordinate efforts at the governmental level (9). However, due to regional disparities in economic development, educational resources, and lifestyle habits, the prevalence patterns and contributing factors of myopia vary significantly across different areas (10, 11). Therefore, region-specific investigations are essential to identify the epidemiological characteristics and key risk factors of myopia at the local level, thereby providing scientific support for the development of targeted and effective prevention strategies.

Several epidemiological studies have been conducted across various regions of China, revealing the prevalence of myopia and associated risk factors among adolescents. For example, data from Hangzhou (12), Weifang (13), Gansu Province (14), and Chengdu (15) have highlighted the prevalence of myopia and key influencing factors such as sex, urban–rural disparities, and school stage. These studies also reflect challenges in myopia control, such as low correction rates and inadequate correction accuracy. However, existing studies face several limitations, including uneven regional coverage, insufficient analysis of refractive parameters, and a lack of targeted intervention strategies. In particular, there is a lack of systematic investigations focusing on the northeastern Sichuan region. This study targets the northeastern Sichuan region, employing a stratified cluster random sampling method to investigate the visual acuity and refractive status of children and adolescents, analyze prevalence and distribution patterns, explore influencing factors, and assess the current status of myopia correction. The findings are expected to fill regional knowledge gaps and provide baseline data for underdeveloped areas in Central and Western China, enrich refractive parameter research to inform integrated prevention and control strategies, and support precise prevention efforts to help achieve national goals for myopia control.

## 2 Research object and methods

### 2.1 Research object

In 2024, a school-based vision and refractive error screening program was conducted in Nanchong City, Sichuan Province, covering 3 districts and 6 counties. This study was implemented within the annual government-supported school vision screening program in collaboration with the local education authority, the “Mingmu” project, and participating schools. A stratified cluster sampling framework was adopted based on administrative region, educational stage, and local screening-resource availability. The final school clusters included 2 kindergartens, 13 primary schools, 15 junior/senior high schools, and 1 vocational high school. Because the survey was school-based rather than age-balanced, and because only two public kindergartens in the study area met the standardized screening conditions and participated in the program, preschool children accounted for a relatively small proportion of the screened population. All participants were enrolled students ranging from preschool to secondary education levels. For stage-based analyses, lower primary referred to Grades 1–3 (approximately 6–9 years), upper primary referred to Grades 4–6 (approximately 9–12 years), and vocational high school referred to secondary vocational education (approximately 15–18 years), which is a category at the same educational level as general high school in the Chinese education system rather than a post-senior-high stage.

For school-type analyses, key schools referred to schools designated by the local education authorities as key or demonstration schools within the corresponding educational stage, whereas “ordinary schools” referred to non-key schools. This classification was applied consistently in the school-type comparison for lower primary, upper primary, junior high, and general high school students.

### 2.2 Methods

All eligible students in the sampled schools were invited to participate in the screening program. Visual acuity testing and non-cycloplegic autorefraction were performed using an electronic intelligent screening system for real-time data collection and automated data storage, following national screening protocols. Screening data were shared between the screening system and parents/guardians to facilitate follow-up management. Schools were informed in advance, and students with habitual refractive correction were instructed to bring their spectacles or other corrective devices on the screening day.

#### 2.2.1 Visual acuity testing

Tests were performed in well-lit environments after a 15-min rest, adhering to the national myopia screening consensus. Uncorrected visual acuity (UCVA), defined as visual acuity measured without any refractive correction, was the core screening indicator in this population-based survey and was used for the diagnosis of poor visual acuity and suspected myopia. Corrected visual acuity (CVA), defined as visual acuity measured while wearing appropriate habitual refractive correction (including frame spectacles, contact lenses, orthokeratology lenses, and other corrective devices), was used only to evaluate the adequacy of correction. Students identified optotypes (starting from 4.0) within 3 s (16).

#### 2.2.2 Refractive error measurement

Uniform autorefractors (WSRMK-8000, Microvision) and LCD visual charts (WSVC-100, Qingda Vision) were used, calibrated daily. Each eye was measured three times, with average values recorded. Non-cycloplegic autorefraction was adopted because this study was conducted as a large-scale school-based screening program, in which cycloplegia was not feasible in routine field implementation. We acknowledge that this approach could not eliminate accommodative bias, particularly in younger children. To reduce misclassification, suspected myopia was defined using both UCVA and SE criteria rather than refractive measurements alone, and the refractive results should therefore be interpreted as screening-based estimates rather than definitive clinical diagnoses.

### 2.3 Quality control

All examiners completed standardized training and certification before fieldwork. During each screening session, 5% of students were randomly selected for repeat testing of all examination items. The allowable inter-test differences were ≤ 1 line for visual acuity and < 0.50 D for spherical equivalent measured by autorefraction. If the error rate in the repeated sample exceeded 5%, the data from that session were not entered into the final statistical analysis. In addition, professional quality inspectors were present on site each day to supervise the screening procedures.

### 2.4 Inclusion and exclusion criteria

Of the 61,012 students initially screened, records were excluded from the final analysis if they met any of the following criteria: (1) incomplete screening data, including missing visual acuity or refractive measurements in one or both eyes; (2) failure to meet the predefined quality-control criteria on repeat testing, defined as a visual acuity discrepancy of >1 line or a spherical equivalent discrepancy of ≥0.50 D between repeated measurements; or (3) reduced visual acuity attributable to organic ocular disease, ocular injury, or other non-refractive causes. All exclusion decisions were independently reviewed by two ophthalmologists to ensure consistency and objectivity. The effective screening rate was defined as the proportion of initially screened students with complete and analyzable data after application of the predefined exclusion criteria.

### 2.5 Indicator definitions

Referring to IMI myopia classification (17), indicators were defined as follows. Non-cycloplegic refraction was adopted for school-based screening feasibility; therefore, the refractive findings, particularly prevalence estimates in preschool and lower primary students, should be interpreted in the context of this screening setting and with caution regarding possible residual accommodative error.

#### 2.5.1 Poor visual acuity

Poor visual acuity was defined based on age-specific UCVA thresholds according to the Expert Consensus on Workflow for Myopia Screening in Children and Adolescents (2019) (16). A participant was classified as having poor visual acuity if either eye met the corresponding UCVA criterion, and severity was graded according to the worse eye. Orthokeratology lens users were directly classified as having poor visual acuity for screening purposes. The grading criteria were as follows: (1) for children aged ≤ 3 years, poor visual acuity was defined as UCVA < 4.8 in either eye, with mild visual impairment defined as UCVA = 4.7, moderate visual impairment as 4.4 ≤ UCVA ≤ 4.6, and severe visual impairment as UCVA ≤ 4.3; (2) for children aged 4–5 years, poor visual acuity was defined as UCVA < 4.9 in either eye, with mild visual impairment defined as UCVA = 4.8, moderate visual impairment as 4.5 ≤ UCVA ≤ 4.7, and severe visual impairment as UCVA ≤ 4.4; and (3) for children aged ≥6 years, poor visual acuity was defined as UCVA < 5.0 in either eye, with mild visual impairment defined as UCVA = 4.9, moderate visual impairment as 4.6 ≤ UCVA ≤ 4.8, and severe visual impairment as UCVA ≤ 4.5.

#### 2.5.2 Suspected myopia

Suspected myopia was defined as UCVA < 5.0 together with spherical equivalent (SE) ≤ −0.50 D in either eye. A participant was classified as having suspected myopia if either eye met the criterion, and severity was graded according to the eye with the more negative refractive error. Orthokeratology lens users were identified through on-site history taking regarding lens wear, together with available pre-orthokeratology refractive information provided by students/parents or prior prescription/clinical records when available. This approximate baseline refractive information was used only to assist in identifying orthokeratology users and their general pre-treatment refractive category, rather than for analyses based directly on measured refractive parameters. In this study, orthokeratology users were retained in screening-based prevalence analyses and correction-status analyses, but were not included in analyses directly based on measured refractive parameters. Low myopia was defined as −3.00 D < SE ≤ −0.50 D, moderate myopia as −6.00 D < SE ≤ −3.00 D, and high myopia as SE ≤ −6.00 D.

#### 2.5.3 Hyperopia and hyperopia reserve

Hyperopia (SE ≥ +2.00D); reserve defined as age-specific physiological hyperopia ( ≤ 6 years: 1.00–3.00D; 7–10 years: 0.25–3.00D).

#### 2.5.4 Astigmatism and anisometropia

Astigmatism was defined as cylindrical diopter (DC) ≤ −0.50 D, and high astigmatism was defined as DC ≤ −2.00 D. Anisometropia was defined as an interocular spherical equivalent (SE) difference of ≥1.00 D or ≥2.00 D.

#### 2.5.5 Spectacle use and correction

Among individuals with suspected myopia, spectacle wearers were those using habitual refractive correction (e.g., frame spectacles, contact lenses, orthokeratology lenses, or other corrective devices). Qualified correction was defined as binocular corrected visual acuity (CVA) ≥ 4.9 and was used only for the evaluation of correction adequacy among corrected students. Accordingly, spectacle compliance rate was defined as the proportion of spectacle wearers who achieved binocular CVA ≥ 4.9 while using habitual refractive correction. Orthokeratology users were included in correction-status analyses as habitual correction users.

### 2.6 Statistical analysis

Data was analyzed using Excel 2016 and SPSS 27.0. Categorical variables were summarized as frequencies and percentages. Chi-square tests were used for between-group comparisons, and Cochran–Armitage trend tests were applied to assess trends across educational stages or grades. Bonferroni correction was used for pairwise comparisons when appropriate. To account for potential confounding factors, multivariable binary logistic regression was performed with suspected myopia as the dependent variable. School grade, gender, regional type, and school level were selected a priori based on epidemiological relevance and data availability and were entered simultaneously into the model to evaluate their independent associations with myopia prevalence. The model was used for association analysis rather than for predictive classification. Odds ratios (ORs) were calculated to quantify the direction and magnitude of association. Because the study used a stratified cluster sampling framework, the potential influence of school-level clustering on the regression estimates should be considered when interpreting the results. A two-sided *P* value < 0.05 was considered statistically significant.

### 2.7 Ethics approval and consent to participate

This study was reviewed and approved by the Medical Ethics Committee of the Second Clinical College of North Sichuan Medical College (Nanchong Central Hospital).(Approval No. 227 [2025]). Because the screening was conducted as part of an annual government-supported school vision screening program, involved routine non-invasive examinations only, posed no more than minimal risk, and was impracticable to implement with individual written informed consent across a very large school-based population, the requirement for written informed consent was waived by the ethics committee. Participant privacy and confidentiality were protected throughout the study. The study was conducted in accordance with the principles of the Declaration of Helsinki.

## 3 Results

### 3.1 Student characteristics and prevalence of poor visual acuity

A total of 61,012 students were initially screened. After exclusion of 1,693 records according to the predefined exclusion criteria, 59,319 students with complete and analyzable data were finally included in this study. Thus, the effective screening rate, defined as the proportion of initially screened students with complete and analyzable data, was 97.23%. The sample had a balanced gender distribution (males: 51.51%, females: 48.49%) and included preschool children (1.61%), primary school students (34.35%), junior high school students (36.55%), general senior high school students (25.71%), and vocational high school students (1.79%), providing a comprehensive overview of visual and refractive conditions across developmental and educational stages.

As shown in Table 1, the detection rate of poor visual acuity (VA) increased significantly with educational stage (χ^2^ = 9434.283, *P* < 0.001), particularly from upper primary grades (Grades 4–6) onward. Kindergarten students had a 55.23% poor VA rate (predominantly mild cases: 29.71%), while lower primary grades showed a slightly reduced rate (45.98%). The rate rose sharply to 72.57% in upper primary grades, with severe cases accounting for 42.15%, and further escalated to 84.91% in junior high (severe cases: 64.77%) and 90.81% in general senior high (severe cases: 76.50%). Vocational senior high students had a lower detection rate (72.45%) with 50.38% severe cases.

**Table 1:** Detection rate and severity of poor visual acuity by educational stage.

Education level	Screened number	Detection rate (%)	95% CI	Mild visual impairment (%)	Moderate visual impairment (%)	Severe visual impairment (%)
Kindergarten	956	55.23	(52.07, 58.39)	29.71^**^	20.92^**^	4.60^**^
Lower primary	11,232	45.98	(45.06, 46.91)	16.36^**^	18.38^**^	11.21^**^
Upper primary	9,144	72.57	(71.66, 73.49)	9.80^**^	20.53^**^	42.15^**^
Junior high	21,679	84.91	(84.44, 85.39)	5.55^**^	14.55^**^	64.77^**^
General high	15,248	90.81	(90.35, 91.26)	3.27^**^	10.94^**^	76.50^**^
Vocational high	1,060	72.45	(69.76, 75.15)	7.45^**^	14.62^**^	50.38^**^
Chi-square trend		8,723.323		2,337.28	529.27	13,792.84
*P*-value		< 0.001		< 0.001	< 0.001	< 0.001

^**^indicates statistically significant pairwise differences between educational-stage groups in *post-hoc* comparisons (Bonferroni-adjusted, where applicable). Mild, moderate, and severe visual impairment were classified according to the age-specific UCVA-based criteria described in the Methods section.

### 3.2 Detection rate and severity of suspected myopia of different grades

Because suspected myopia showed a progressive grade-related increase in both prevalence and severity, a grade-level analysis was used to provide a more detailed description of this pattern. The detection rate of suspected myopia differed significantly by grade (χ^2^ = 14,551.612, *P* < 0.001), showing a distinct upward trend from kindergarten through the senior secondary stage, including both general high school and vocational high school students. Rates ranged from 14.20%−23.24% in kindergarten, increased progressively from 19.04% (Grade 1) to 74.21% (Grade 6) in primary school, and further rose to 77.18%−85.79% in junior high and 88.40%−89.78% in general senior high; vocational high school students had lower rates (65.49%−68.29%) (Table 2).

**Table 2:** Grade-specific detection rates and severity distribution of suspected myopia.

Grade	Number of students	Detection rate (%)	95% CI (%)	Low degree (%)	Moderate degree (%)	High degree (%)
Kindergarten 1	66	23.24^**^	18.3–28.18	18.66	3.87	0.70^**^
Kindergarten 2	55	16.82^**^	12.74–20.89	15.29	1.22	0.31^**^
Kindergarten 3	49	14.20^**^	10.5–17.9	12.17	2.03	0.00^**^
Grade 1	830	19.04^**^	17.88–20.21	17.00	1.86	0.18^**^
Grade 2	1,170	31.53^**^	30.03–33.02	26.70	4.15	0.57^**^
Grade 3	1,443	45.64^**^	43.9–47.37	37.63	7.46	0.54^**^
Grade 4	1,634	56.23^**^	54.42–58.03	42.50	12.53	1.20^**^
Grade 5	2,233	68.90^**^	67.3–70.49	43.94	22.31	2.59^**^
Grade 6	2,224	74.21^**^	72.64–75.77	40.91	29.43	3.64^**^
Junior high 1	6,069	77.18^**^	76.26–78.11	37.34	33.51	6.32^**^
Junior high 2	6,042	81.96^**^	81.08–82.84	34.77	37.72	9.40^**^
Junior high 3	5,528	85.79^**^	84.93–86.64	33.30	40.75	11.69^**^
General high 1	5,220	88.40^**^	87.58–89.22	28.47	43.08	16.66^**^
General high 2	5,549	89.04^**^	88.26–89.82	26.44	44.80	17.75^**^
General high 3	2,793	89.78^**^	88.71–90.84	23.75	44.81	21.18^**^
Vocational high 1	336	68.29^**^	64.17–72.42	34.35	27.85	6.10
Vocational high 2	372	65.49^**^	61.57–69.41	32.92	24.82	7.75
Chi-square (χ^2^)		14,551.612				3,441.592
Value		< 0.001				< 0.001

Number of students refers to the total number of screened students in each grade of the final analytic sample. Detection rate (%) refers to the proportion of students in each grade who met the screening criteria for suspected myopia. Low-, moderate-, and high-myopia percentages were calculated using the total number of students in each grade as the denominator. Grade-level analysis was used in Table 2 to better illustrate the progressive pattern of suspected myopia prevalence and severity across school progression. ^**^indicates statistically significant pairwise differences between grade groups in *post-hoc* comparisons (Bonferroni-adjusted, where applicable).

**Figure 1:**
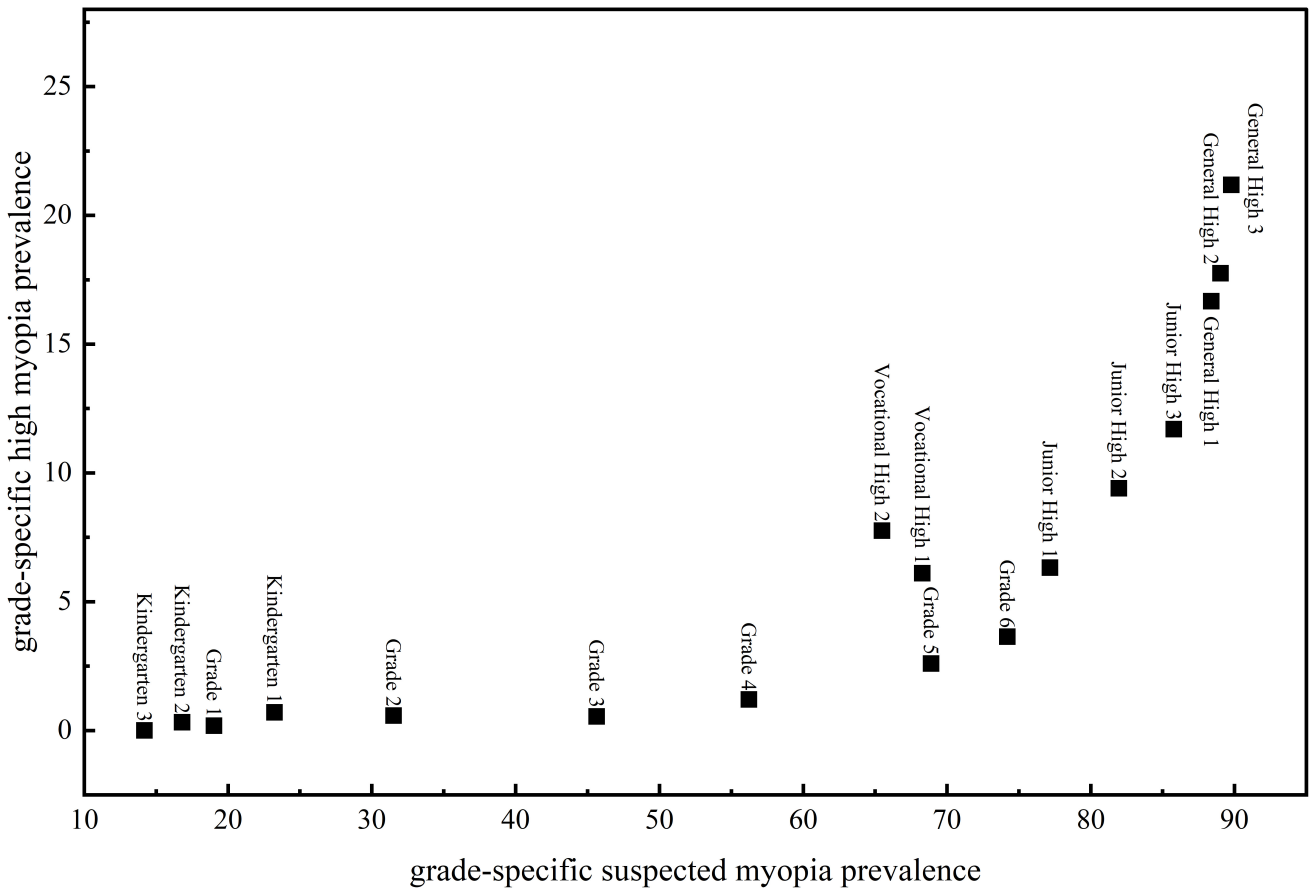
Scatter plot showing the grade-specific relationship between suspected myopia prevalence and high-myopia prevalence. Each point represents one grade group. The x-axis indicates grade-specific suspected myopia prevalence (%), and the y-axis indicates grade-specific high-myopia prevalence (%).

Myopia severity also varied by grade (χ^2^ = 3,441.592, *P* < 0.001). Low myopia was the most common across all grades, but the proportions of moderate and high myopia increased with advancing grade—e.g., 23.75% low, 44.81% moderate, and 21.18% high myopia in Grade 3 of vocational high school. These results confirm that both myopia prevalence and severity rise with grade level. Figure 1 shows the grade-specific relationship between suspected myopia prevalence and high-myopia prevalence, with each point representing one grade group. The figure shows that grade groups with higher suspected myopia prevalence also tended to have higher high-myopia prevalence. This descriptive pattern suggests that the burden of high myopia increased alongside overall myopia prevalence across grade groups.

### 3.3 Gender differences in detection rate and severity of suspected myopia

As shown in Table 3, overall suspected myopia detection rates differed significantly by gender (χ^2^ = 217.636, *P* < 0.001). Females consistently had higher rates than males from kindergarten onward, with the gender disparity becoming more pronounced with advancing grade; differences were statistically significant from Grade 2 to senior high Grade 3. For example, myopia prevalence was 29.91% in males vs. 33.26% in females in Grade 2 (χ^2^ = 4.810, *P* < 0.05), rising to 87.64% in males vs. 91.82% in females in senior high Grade 3 (χ^2^ = 75.25, *P* < 0.001).

**Table 3:** Suspected myopia rates and severity by gender and grade.

Grade	Myopia (Boys)	Myopia (Girls)	*P*	Low (Boys)	Low (Girls)	*P*	Moderate (Boys)	Moderate (Girls)	*P*	High (Boys)	High (Girls)	*P*
Kindergarten 1	22.88	23.66	0.875	18.95	18.32	0.891	2.61	5.34	0.235	1.31	0.0	0.548
Kindergarten 2	19.16	14.38	0.247	17.96	12.5	0.17	1.2	1.25	1	0.0	0.63	0.983
Kindergarten 3	13.98	14.47	0.897	12.37	11.95	0.906	1.61	2.52	0.834	0.0	0.0	^*^
Grade 1	18.86	19.25	0.746	16.7	17.34	0.579	1.94	1.76	0.662	0.22	0.15	0.861
Grade 2	29.91	33.26	0.028	25.27	28.24	0.041	3.91	4.41	0.445	0.63	0.5	0.617
Grade 3	42.65	48.8	< 0.001	35.51	39.88	0.011	6.71	8.26	0.096	0.43	0.65	0.398
Grade 4	53.0	59.74	< 0.001	40.66	44.5	0.036	11.02	14.16	0.011	1.32	1.08	0.551
Grade 5	65.37	72.76	< 0.001	43.03	44.93	0.275	19.56	25.31	< 0.001	2.66	2.52	0.8
Grade 6	70.23	78.24	< 0.001	40.25	41.57	0.463	27.19	31.7	0.007	2.72	4.57	0.007
Junior high 1	73.51	81.23	< 0.001	35.9	38.93	0.005	31.68	35.53	< 0.001	5.92	6.76	0.123
Junior high 2	79.15	84.87	< 0.001	32.64	36.97	< 0.001	36.75	38.74	0.078	9.71	9.08	0.359
Junior high 3	83.96	87.74	< 0.001	32.23	34.45	0.059	40.4	41.12	0.556	11.29	12.1	0.313
General high 1	86.69	90.13	< 0.001	27.09	29.86	0.018	42.72	43.45	0.575	16.81	16.51	0.758
General high 2	87.32	90.74	< 0.001	25.45	27.43	0.077	43.42	46.17	0.029	18.45	17.05	0.148
General high 3	87.64	91.82	< 0.001	23.21	24.28	0.484	43.33	46.23	0.104	21.04	21.32	0.847
Vocational high 1	66.77	71.34	0.304	32.32	38.41	0.179	28.05	27.44	0.887	6.4	5.49	0.689
Vocational high 2	62.71	70.09	0.073	30.79	36.45	0.164	23.45	27.1	0.328	8.47	6.54	0.404
Total	67.46	73.01	< 0.001	30.83	33.38	< 0.001	28.22	30.9	< 0.001	8.37	8.64	0.243

Percentages for myopia, low myopia, moderate myopia, and high myopia are presented separately for boys and girls within each grade. The χ^2^ and *P* values represent comparisons between boys and girls within the same grade category. ^*^indicates that the statistical comparison was not applicable or not estimable because of sparse or zero cell counts.

Regarding severity, significant gender differences were noted in low and moderate myopia overall (low: χ^2^ = 44.188, moderate: χ^2^ = 51.221; both *P* < 0.001), with specific grade-level differences in low and high myopia. Low myopia was the most common in all genders, with females having slightly higher proportions; the proportions of moderate and high myopia in females increased with grade. These findings indicate that gender is a key factor in myopia development, with females at higher risk across all grades.

### 3.4 Regional and school-type differences in suspected myopia across education stages

Suspected myopia detection rates differed significantly by region (χ^2^ = 2490.783, *P* < 0.001), ranging from 46.41% in Langzhong City to 80.49% in Nanbu County. Significant regional differences were also observed in myopia severity (low: χ^2^ = 131.538, moderate: χ^2^ = 1167.958, high: χ^2^ = 611.543; all *P* < 0.001): low myopia was most common in Yingshan County (33.82%) and least in Langzhong City (20.63%); moderate myopia peaked in Xichong County (38.23%) and was lowest in Peng'an County (19.13%); high myopia was highest in Nanbu County (11.78%) and lowest in Peng'an County (3.58%).

Beyond inter-regional disparities, urban-rural and school-type differences also emerged as key determinants of myopia prevalence, as presented in Table 4. Data showed urban students had significantly higher myopia rates than township peers across all educational stages (*P* < 0.001). Regarding school type, schools classified as key schools by the local education authorities had higher myopia rates than ordinary schools at the primary stage; however, this trend reversed in junior and general senior high schools, with a pronounced difference in general senior high (*P* < 0.001). These results indicate significant regional and school-type differences in suspected myopia prevalence across educational stages.

**Table 4:** Suspected myopia rates of different regional and school-type by education stages.

	Region						School type					
Education level	Urban (*n*)	Urban (%)	Township (*n*)	Township (%)	χ^2^	*P*	Ordinary (*n*)	Ordinary (%)	Key (*n*)	Key (%)	χ^2^	*P*
Lower primary	3,205	31.12	238	25.51	12.668	< 0.001	3,021	30.38	422	32.79	3.120	0.077
Upper primary	5,468	67.47	623	59.90	23.743	< 0.001	5,293	66.55	798	67.00	0.094	0.759
Junior high	16,126	82.32	1,513	72.43	121.785	< 0.001	13,063	81.79	4,576	80.17	7.313	0.007
General high	12,451	89.44	1,111	83.72	40.274	< 0.001	7,758	90.06	5,804	87.49	25.248	< 0.001

Key refers to schools designated by the local education authorities as key or demonstration schools, and “Ordinary” refers to non-key schools. This classification was applied consistently to lower primary, upper primary, junior high, and general high school students; kindergarten and vocational high school were not included in the school-type comparison.

### 3.5 Distribution of spectacle types among students at different educational stages

Framed eyeglasses remain the predominant corrective option across all educational stages (43.03%), and their usage proportion increased progressively: from 1.15% in kindergarten, to 67.29% in general senior high. Orthokeratology lens usage was relatively low across all stages (slightly higher in upper primary), while other corrective lenses were rarely used.

Further analysis showed significant differences in spectacle-wearing and compliance rates across educational stages (*P* < 0.001, Table 5). Notably, the spectacle-wearing rate among students with suspected myopia rose progressively from 0.59% (kindergarten) to 75.57% (senior high), reflecting growing vision correction demand with advancing grades. However, compliance rates, defined in this study as the proportion of spectacle wearers with binocular corrected visual acuity (CVA) ≥ 4.9, did not increase proportionally (63.38% in lower primary; 74.71% in senior high), indicating unmet needs in standardized vision correction. Gender differences were significant in spectacle-wearing rates (*P* < 0.001) but not in compliance rates (*P* = 0.098). Although spectacle-wearing rates increased with educational stage, qualified correction did not improve proportionally.

**Table 5:** Eyeglass wearing status and correction qualification among students with suspected myopia.

Category	Wearing glasses				Qualified correction			
	*n*	%	χ^2^	*P*	*n*	%	χ^2^	*P*
School stage			4,792.716	< 0.001			313.159	< 0.001
Kindergarten	1	0.59			0.00	0.00		
Primary (Lower)	680	19.75			431	63.38		
Primary (Upper)	2,756	45.25			1,601	58.09		
Junior high	11,267	63.88			7,875	69.89		
General high	10,249	75.57			7,657	74.71		
Vocational high	256	36.16			170	66.41		
Gender			106.882	< 0.001			2.741	0.098
Male	11,972	58.08			8,482	70.85		
Female	13,237	63.03			9,252	69.89		

Spectacle-wearing rate, Number of individuals with suspected myopia wearing habitual refractive correction / Total number of individuals with suspected myopia. Spectacle compliance rate, Number of individuals with binocular corrected visual acuity (CVA) ≥ 4.9 while wearing habitual refractive correction / Number of individuals with suspected myopia who wear habitual refractive correction.

### 3.6 Distribution of astigmatism, anisometropia and hyperopia by educational stage

Statistical analysis (Table 6) revealed distinct variations in astigmatism (DC ≤ −0.50D and DC ≤ −2.00D) across educational stages (*P* < 0.001), with males showing higher rates than females (DC ≤ −0.50D: P = 0.029; DC ≤ −2.00D: *P* < 0.001). Astigmatism (DC ≤ −0.50D) increased progressively from 72.38% (kindergarten) to 84.21% (general senior high), while high astigmatism (DC ≤ −2.00D, a risk factor for amblyopia) remained relatively uncommon but also rose with grade. Anisometropia (SE ≥ 1.0D and SE ≥ 2.0D) showed notable disparities by stage (*P* < 0.001), with females having a higher prevalence of SE ≥ 1.0D (*P* < 0.001) and increasing with grade. A detailed analysis of the suspected myopia population showed stage–related differences in both conditions (*P* < 0.001), with significant gender disparities in astigmatism (*P* < 0.001) and SE ≥ 2.0D anisometropia (*P* = 0.022). Their prevalence exceeded that in the general population.

**Table 6:** Distribution of astigmatism and anisometropia by school stage and gender.

Category	Astigmatism								Anisometropia							
	DC ≤ −0.50D		χ^2^	*P*	DC ≤ −2.00D		χ^2^	*P*	SE ≥1.00D		χ^2^	*P*	SE ≥2.00D		χ^2^	*P*
	*n*	%			*n*	%			*n*	%			*n*	%		
School stage			383.247	< 0.001			215.951	< 0.001			1,165.134	< 0.001			575.495	< 0.001
Kindergarten	692	72.38			41	4.29			218	22.80			75	7.85		
Primary (Lower)	8,521	75.86			711	6.33			2,022	18.00			545	4.85		
Primary (Upper)	7,115	77.81			648	7.09			2,410	26.36			738	8.07		
Junior high	17,504	80.74			1,948	8.99			7,126	32.87			2,565	11.83		
General high	12,840	84.21			1,626	10.66			5,420	35.55			1,937	12.70		
Vocational high	73.87	73.90			71	6.70			301	28.40			102	9.62		
Gender			4.749	0.029			61.527	< 0.001			17.415	< 0.001			0.585	0.444
Male	24,550	80.35			2,865	9.38			8,781	28.74			3,043	9.96		
Female	22,905	79.63			2,180	7.58			8,716	30.30			2,919	10.15		

DC ≤ −0.50 D indicates astigmatism, and DC ≤ −2.00 D indicates high astigmatism.

Regarding hyperopia and reserve, hyperopia prevalence declined from 2.09% (kindergarten) to 0.82% (general senior high) (P < 0.001). Younger students had abundant reserve: 21.68% in Grade 1, decreasing progressively to 8.82% in Grade 3.

These findings underscore the need to monitor astigmatism and anisometropia, with gender-tailored interventions prioritizing younger students to mitigate myopia risk and impair visual quality.

### 3.7 Logistic regression analysis of confounding factors associated with myopia

A multivariable binary logistic regression model was employed to evaluate the independent associations of school grade, gender, regional type, and school level with myopia prevalence after mutual adjustment for these potential confounding factors. As presented in Table 7, grade (OR = 1.404, *P* < 0.001), gender (OR = 1.363, *P* < 0.001), and regional type (OR = 1.637, *P* < 0.001) were positively associated with myopia, while school level (OR = 0.753, *P* < 0.001) showed a negative association. These findings indicate that, after adjustment for the other variables included in the model, higher school grade, female sex, urban residency, and attendance at regular schools were independently associated with a higher risk of myopia.

**Table 7:** Logistic regression analysis of confounding factors associated with myopia.

Variable	*B*	S.E.*(B)*	Wald	*P*	OR
Grade	0.339	0.003	10,281.030	< 0.001	1.404
Gender	0.310	0.021	229.065	< 0.001	1.363
Area Type	0.493	0.035	202.974	< 0.001	1.637
School Level	−0.283	0.025	129.906	< 0.001	0.753
Constant	−1.193	0.032	4,027.681	< 0.001	0.130

Gender was coded as female vs. male; Area type as urban vs. township; School level as ordinary school vs. key school.

## 4 Discussion

This study, based on a large-scale school-based vision and refractive error screening program in Nanchong City in 2024, provides a comprehensive overview of visual acuity and refractive status among children and adolescents in northeastern Sichuan. Using an electronic intelligent screening system with standardized procedures and quality control, the study identified marked educational-stage and grade-related differences in poor visual acuity, suspected myopia, correction status, and other refractive abnormalities. These findings provide important regional evidence for myopia prevention and control in an underrepresented area of southwestern China.

A major finding of this study is the clear increase in poor visual acuity and suspected myopia with advancing educational stage and grade. Poor visual acuity reached 90.81% in general senior high school students, and suspected myopia approached 90% in the same stage, with a parallel increase in myopia severity and the proportion of high myopia. This age- and grade-related pattern is broadly consistent with other large-scale screening studies conducted in Hangzhou, Weifang, Gansu, and Chengdu, all of which reported marked increases in myopia prevalence with advancing school stage (12–15). Compared with these regions, the present findings further highlight the substantial burden of myopia in northeastern Sichuan and may reflect differences in educational pressure, near-work burden, and outdoor exposure reported in previous studies (18, 19).

The study also identified clear gender differences in suspected myopia. Female students had consistently higher myopia prevalence than male students, and this difference became more pronounced in the higher grades. This finding is in line with previous reports suggesting that female students may be at greater risk of myopia (20, 21). Although the present study did not directly measure behavioral or physiological mechanisms, the observed difference may be related to variation in visual habits, outdoor activity, or developmental factors reported in prior studies (20, 21). These possible explanations should, however, be interpreted with caution because such variables were not directly assessed in the present dataset.

Another important result is the substantial regional and school-context heterogeneity in suspected myopia prevalence. Significant differences were observed across counties, with prevalence ranging from 46.41% in Langzhong City to 80.49% in Nanbu County. Urban students also had consistently higher myopia prevalence than township students, and school-type differences varied by educational stage. In particular, students in ordinary schools showed higher myopia prevalence than those in key schools in junior high and general senior high school, whereas the pattern was less pronounced or reversed at earlier stages. These findings indicate that myopia burden in northeastern Sichuan is not evenly distributed across contexts. The observed disparities may reflect broader differences in urbanization, educational pressure, access to eye-health services, prevention practices, and parental supervision, but these factors were not directly measured in this study and therefore should not be interpreted as confirmed explanatory variables. Similar urban–rural and contextual disparities have been reported in previous studies (12, 14, 21–23). Taken together, these findings support the need for targeted myopia prevention strategies that are sensitive to both regional and school-type differences.

The correction-related findings also have practical importance. Frame spectacles remained the predominant correction method, and spectacle-wearing rates increased progressively with educational stage. However, qualified correction did not improve proportionally with increasing spectacle use. This suggests that greater use of correction does not necessarily translate into adequate correction quality. The observed gap between increasing spectacle-wearing rates and suboptimal correction compliance may reflect several practical barriers, including poor adaptation to spectacles, inaccurate or outdated prescriptions, insufficient parental follow-up, and financial or service-access constraints affecting timely replacement, refitting, and regular eye examinations. Because these factors were not directly measured in the present study, they should be interpreted as possible explanations rather than measured determinants. Previous studies have also emphasized that continued care, appropriate prescription, and follow-up are essential for improving visual outcomes in school-aged children (24–26). Further investigation of spectacle-wearing behavior and adequacy of correction across regions and school types would be valuable to better characterize disparities in access to and quality of vision correction.

In addition to myopia prevalence, this study found a high prevalence of astigmatism and anisometropia, particularly among students with suspected myopia, and both conditions tended to increase with advancing school stage. These findings suggest that refractive abnormality in this population is not limited to spherical myopia alone. From a clinical perspective, the coexistence of astigmatism and anisometropia may complicate refraction, reduce visual quality, and increase the difficulty of achieving adequate correction, highlighting the importance of careful refractive assessment, appropriate spectacle fitting, and follow-up management (27). At the same time, hyperopia reserve declined progressively in lower-grade students, suggesting that early refractive development in younger children deserves close monitoring, particularly in the context of school-based screening and prevention.

The present findings have several implications for public health practice. Given the marked increase in myopia burden from upper primary school onward, preventive efforts should focus particularly on upper primary, junior high, and general senior high school students. School-family-medical collaboration may be especially important in these stages to promote appropriate outdoor activity, reduce excessive near-work burden, improve eye-use habits, and enhance follow-up for students who already require correction. In addition, the observed contextual disparities suggest that resource allocation for screening, refraction, and follow-up services should take account of regional and school-type differences rather than assuming a uniform burden across all student groups (22, 23). Whether multifocal or progressive lenses may provide additional benefit for students with these refractive abnormalities should be investigated in future studies.

This study has several limitations. First, the cross-sectional design does not allow causal inference and cannot characterize the dynamic progression of myopia over time. Second, the study lacked detailed behavioral variables such as outdoor activity duration, near-work burden, and screen exposure, which limited our ability to explain the mechanisms underlying the observed differences. Third, although clear regional and school-type disparities were identified, contextual variables such as socioeconomic status, school-level prevention practices, and healthcare accessibility were not directly measured, so these factors can only be discussed as possible contributors rather than confirmed determinants. Fourth, because the study used a stratified cluster sampling framework, school-level clustering was not explicitly modeled in the primary logistic regression analysis, and formal multicollinearity diagnostics were not separately reported. Therefore, the regression estimates should be interpreted with appropriate caution. An additional limitation relates to the use of non-cycloplegic autorefraction in this large-scale school-based screening setting. Although this approach was operationally feasible and consistent with routine field implementation, it could not eliminate accommodative bias, particularly in preschool and lower primary students. As a result, myopia-related outcomes in younger age groups may have been overestimated, while hyperopia and hyperopia reserve may have been underestimated or misclassified. We attempted to reduce this bias through standardized examiner training, instrument calibration, triplicate measurements, repeat testing, and the combined use of UCVA and SE criteria, but these measures could only reduce, rather than remove, residual bias. Similarly, orthokeratology users were retained in screening-based prevalence and correction analyses but excluded from analyses based directly on measured refractive parameters, because orthokeratology alters corneal curvature and may affect refractive measurement. These findings in younger age groups should therefore be interpreted with appropriate caution (28).

Despite these limitations, this study provides the first large-scale, systematic description of visual acuity and refractive status among children and adolescents in northeastern Sichuan. By documenting the substantial burden of poor visual acuity, suspected myopia, correction gaps, and contextual disparities in this region, the study offers useful baseline evidence for the development of more targeted local myopia prevention and control strategies. Future studies should incorporate longitudinal follow-up, richer behavioral and contextual variables, and more detailed evaluation of correction services to further clarify the determinants and consequences of refractive problems in this population (26, 29).

## 5 Conclusion

This large-scale vision and refractive screening of school-aged youths in Nanchong City revealed a significant increase in the prevalence of vision impairment and suspected myopia with advancing educational stage, alongside a continuous rise in the proportion of high myopia. Marked disparities were evident between urban and rural schools, with urban students having significantly higher myopia rates. In the primary stage, key schools had higher myopia rates compared to ordinary schools. Conversely, in junior and senior high stages, ordinary schools showed higher myopia rates than key schools, especially in senior high, where the difference was pronounced. Female sex, higher grade level, urban residency, and attendance at ordinary schools were identified as factors independently associated with myopia. Astigmatism and anisometropia were highly prevalent within the myopic population, highlighting the need for focused attention to spectacle fitting and follow-up management. Although frame glasses remain the predominant correction method and spectacle wearing rates increased with educational stage, compliance with proper spectacle use requires further improvement. This study provides robust data to support vision health monitoring and targeted myopia control strategies for children and adolescents in northeastern Sichuan. It is recommended that future efforts incorporate longitudinal follow-up, intervention assessment, and multi-center collaboration to continuously optimize adolescent visual health management systems and advance both regional and national myopia prevention goals.

## Data Availability

The data analyzed in this study is subject to the following licenses/restrictions: The dataset involves sensitive health information of minors (adolescent visual status data). To protect the privacy and rights of participants, the dataset is not publicly available. Access to the data is restricted to the research team and relevant ethics oversight bodies, in compliance with local data protection regulations and the ethical approval of this study. Requests to access these datasets should be directed to Zhe Li, E-mail: lz09132025@163.com.
